# Population Dynamics of Bigeye Grunt *Brachydeuterus auritus* (Valenciennes, 1831) in the Coastal Waters of Sierra Leone: A Near-Threatened Species on the IUCN Red List

**DOI:** 10.3390/biology14081037

**Published:** 2025-08-12

**Authors:** Guoqing Zhao, Chunlei Feng, Hewei Liu, Taichun Qu, Ruiliang Fan, Ivorymae C. R. Coker, Lahai Duramany Seisay, Hongliang Huang, Lingzhi Li

**Affiliations:** 1 East China Sea Fisheries Research Institute, Chinese Academy of Fishery Sciences, Shanghai 200090, China; zgq617717@163.com (G.Z.); fengmaster@126.com (C.F.); hwliu77@126.com (H.L.); qtc8810@163.com (T.Q.); ruiliangfan@163.com (R.F.); ecshhl@163.com (H.H.); 2 Key Laboratory of East China Sea and Oceanic Fishery Resources Exploitation, Ministry of Agriculture and Rural Affairs, Shanghai 200090, China; 3 Ministry of Fisheries and Marine Resources, Youyi Building, Freetown, Sierra Leone; ivorymae007m@gmail.com (I.C.R.C.); sldmaster@126.com (L.D.S.)

**Keywords:** *Brachydeuterus auritus*, biological characteristics, stock assessment, LBB, GAM, tempo-spatial distribution, Sierra Leone

## Abstract

Bigeye grunt (*Brachydeuterus auritus*) is a dominant fish species and mostly a major target species in both artisanal and industrial fisheries in the coastal waters of Sierra Leone. It was listed as near threatened in 2015 by the International Union for Conservation of Nature (IUCN) Red List. Although this species has been repeatedly assessed as overexploited by the Fishery Committee for the Eastern Central Atlantic (CECAF) in the majority of its range in the Eastern Central Atlantic, there have never been studies of stock assessment in the coastal waters of Sierra Leone. We conducted a study on the population dynamics of bigeye grunt in the coastal waters of Sierra Leone, which is crucial for completing the resource status of this species in the Eastern Central Atlantic. The results showed that the bigeye grunt had a wide distribution in the coastal waters of Sierra Leone, with significant spatiotemporal variation characteristics in biomass and abundance. The growth parameters of bigeye grunt varied across different months, but all *E* values were below 0.5, indicating that no overfishing occurred. These findings were further corroborated by the results of the Length-Based Bayesian Biomass Estimation method (LBB). The results of the Generalized Additive Model (GAM) show that there is a certain nonlinear relationship between the resource abundance of the bigeye grunt and both environmental factors and geographical locations, among which the influence of latitude is the greatest. This study posits that the bigeye grunt in Sierra Leone’s coastal waters exhibits moderate exploitation potential. The findings are anticipated to provide a scientific framework for informing evidence-based management strategies for this fishery resource.

## Simple Summary

Bigeye grunt (*Brachydeuterus auritus*) is a major bycatch fish species in both artisanal and industrial fisheries in coastal waters of Sierra Leone. It was listed as near threatened in 2015 by the International Union for Conservation of Nature (IUCN) Red List. However, there has never been a study of stock assessment and spatiotemporal distribution for this species in the coastal waters of Sierra Leone. In this case, we studied both the population dynamics and stock assessment of bigeye grunt in this area. We found that the bigeye grunt is widely distributed and a dominant species in the coastal waters of Sierra Leone, demonstrating potential for exploitation and showing no signs of overfishing. Both environmental factors and geographical locations exert a certain degree of nonlinear influence on their resource abundance. Our study is of great significance for understanding the population dynamics of bigeye grunt in the coastal waters of Sierra Leone.

## 1. Introduction

The bigeye grunt *Brachydeuterus auritus* (Valenciennes, 1832), widely distributed in the tropical and subtropical waters of the Eastern Atlantic Ocean, serves as a primary target species for both artisanal fisheries and subsistence fishing operations throughout its entire range [1,2]. Recognized for its cost effectiveness and high protein content, throughout its distribution area, it serves as a primary source of animal protein for numerous coastal households [2] and is further identified as an optimal provider of premium-quality protein in infant diets [3], making substantial contributions to regional food security and nutritional sustainability. In 2015, the International Union for Conservation of Nature (IUCN) listed it as “near threatened,” underscoring the persistent exploitation pressures confronting this species [1]. Although the Eastern Central Atlantic Fisheries Commission (CECAF) has repeatedly assessed that the bigeye grunt stock is overexploited in its main range [1,4], the long-term absence of background data on the resource in Sierra Leone’s Exclusive Economic Zone (EEZ) has led to a significant geographic blindness in the assessment of the stock’s sustainability in this area.

Systematic research on bigeye grunt remains limited, with existing studies primarily focused on population life-history parameters (such as growth rate, mortality rate, body length at first maturity, etc.), as well as the length–weight relationship and growth equations [5,6,7,8,9] while lacking investigations into resource distribution and stock assessment. In Sierra Leone, fisheries contribute 9.4% of the national Gross Domestic Product (GDP), with fish accounting for 75% of dietary animal protein intake among the population [10]. However, historical, economic, and political constraints have resulted in persistent gaps in systematic scientific fishery surveys, leading to a critical paucity of science-based fisheries research and a disconnect between fishery production and management frameworks [10]. Consequently, the spatiotemporal distribution and stock assessment of coastal bigeye seabream populations, regulated by seasonal upwelling dynamics in Sierra Leonean waters, have never been documented. Traditional assessment methodologies relying on catch statistics (e.g., catch per unit effort, CPUE) or length–frequency data analysis fail to capture the species’ spatially and temporally heterogeneous distribution patterns within complex hydrographic systems. Furthermore, Bayesian stock assessment models designed for data-limited fisheries remain unvalidated in such ecosystems, indicating substantial potential for refining precision management strategies in this maritime zone.

Sierra Leone features a typical tropical monsoon climate, characterized by high temperatures year round and distinct dry and rainy seasons. During the rainy season, precipitation increases significantly while solar radiation weakens, with the opposite pattern observed in the dry season. Seasonal variations in rainfall and solar radiation exert a significant influence on fluctuations in seawater temperature and salinity in coastal areas [11]. The coastal region of Sierra Leone is recognized as one of the most vulnerable areas to global climate change [12]. Environmental changes have substantial impacts on the distribution and abundance of fishery resources; however, relevant studies in this region remain limited. Against this backdrop, the present study employs the Generalized Additive Model (GAM) to investigate the effects of various factors on the resource abundance of bigeye grunt, and it is believed that this research holds significant importance. The GAM is currently one of the most widely used methods in the field of fisheries, primarily applied to address nonlinear relationships between response variables and multiple explanatory variables [13,14,15,16].

This study presents the first systematic assessment of the population dynamics of the bigeye grunt in Sierra Leone’s coastal waters, thereby addressing the knowledge gap regarding stock assessment for this species in the Eastern Central Atlantic. Meanwhile, this study also explores the impacts of environmental factors and geographical variables on the resource abundance of bigeye grunt, thereby deepening people’s understanding of this species. The findings hold significant scientific value for regional fisheries management. The objectives of this study are as follows: (1) to quantify spatiotemporal variation patterns in biomass and abundance; (2) to assess resource exploitation potential through growth parameter analysis and exploitation rate thresholds; (3) to validate population status using the Length-Based Bayesian Biomass Estimation (LBB) method; and (4) to examine how changes in explanatory variables affect resource abundance.

The novelty of this study manifests in two dimensions: methodological integration and practical implications. Methodologically, coupling length–frequency data with the LBB model has overcome resource assessment bottlenecks in data-limited marine areas. Managerially, the confirmation of moderate exploitation potential in Sierra Leonean populations—coupled with observed spatiotemporal volatility—necessitates establishing dynamic monitoring systems to mitigate localized stock collapse risks. These findings provide Regional Fisheries Management Organizations (RFMOs) in the Eastern Central Atlantic with scientific foundations for designing eco-unit-based quota allocation mechanisms while simultaneously offering a regional paradigm for sustainable exploitation of data-poor coastal fisheries globally.

## 2. Materials and Methods

### 2.1. Surveys

**Figure 1:**
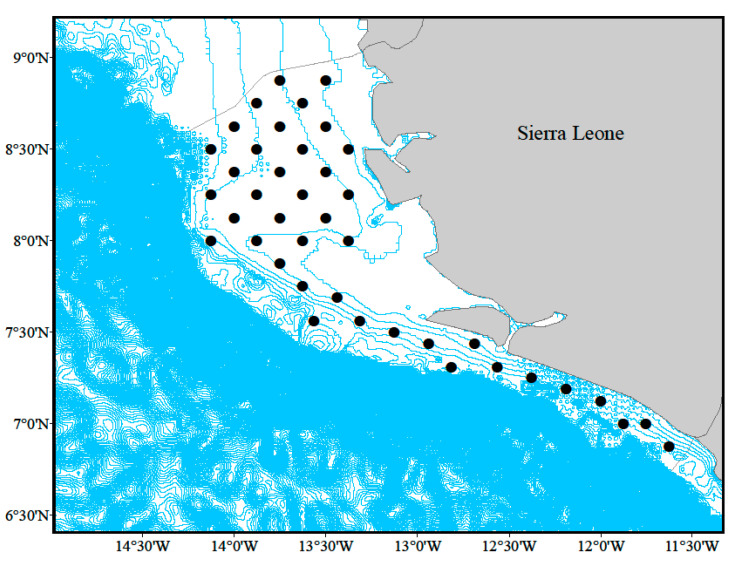
The sample area of survey stations (black dots mark sample station positions; the blue contour lines show 10 m depth intervals).

Six demersal trawl surveys on the fishery research vessel “ZYK212” were conducted in the coastal waters of Sierra Leone, which covered the areas of 6°52′~8°52′ N, 11°37′~13°45′ W, with a total of 41 stations in each in September (Sep) and October (Oct) 2019, December 2020 (Dec), and January (Jan), April (Apr), and May (May) 2021, and the depth range of the survey stations was 11–60 m (Figure 1). The survey gear was a single-vessel, sleeved, single-bladder trawl with a perimeter mesh of 480 mesh, and the main scale of the gear was 144 m × 90.13 m. The operating time per station was 45 to 75 min, and the trawling speed was 3.5 knots.

To estimate swept area, GPS was used to record the towed distance between initial bottom contact and winch retrieval. The mean seabed width covered by the trawl was 15.012 m, determined through pre-survey tests at an average vessel speed of 3.5 knots. Vertical profiles of temperature and salinity were collected at 1 m depth intervals using a Seabird SBE9/11plus CTD system (Sea–Bird Scientific, Bellevue, WA, USA) equipped with integrated sensors.

For each station, all samples of bigeye grunt were measured if the number of samples was less than 30, and 50 samples were measured if the number of samples was greater than 30, with a total of 6642 samples measured (Table 1). Samples were collected, and biological measurements were made in accordance with the Code of Practice for Marine Surveys (CPMS). Measurements included sex, body length, weight, and gonadal maturity, with an accuracy of 1 mm for body length and 0.1 g for weight. According to the visual observation method to record the gonadal maturity and feeding intensity, the gonads were divided into 6 stages, which were expressed as I~VI, and in this study, the grades IV~VI were recognized as gonadal maturity [17].

### 2.2. Data Analysis

Abundance was calculated by dividing the swept area by the total catch per valid trawl (unit: ind/km^2^). Biomass was defined as the weight of bigeye grunt per square kilometer (unit: kg/km^2^). Probability of occurrence, contribution to the total weight, and contribution to the total number refer to the occurrence probability, weight proportion, and quantity proportion of the bigeye grunt among all survey stations, respectively.

The length–weight correlation for bigeye grunt was mathematically quantified using the equation [18]:

*W* = *aL*^*b*^ (1)

where *W* denotes body weight (g), *L* represents standard length (cm), *a* is the coefficient, and *b* is the allometric exponent. The exponent *b* serves as a biological indicator, revealing growth pattern disparities between somatic mass development and linear elongation [19]. Isometric growth occurs at *b* ≈ 3, while deviations from this value signify allometric growth: positive allometry (*b* > 3) indicates disproportionate weight gain relative to length, whereas negative allometry (*b* < 3) suggests length-dominated growth [20,21].

The growth dynamics of bigeye grunt were modeled using the Von Bertalanffy equation [22]:

*L_t_* = *L*_inf_ (1 − e^−*K*(*t* − *t*^_0_^)^) (2)

where *L_t_* is the fish length (cm) at time *t*, *L*_inf_ is the asymptotic standard length, *K* is the growth coefficient (year)^−1^, and *t*_0_ is the theoretical age at length zero. The *L*_inf_ and *K* parameters were calculated through electronic length–frequency analysis (ELEFAN I) implemented in FAO’s FISAT II software (Version 1.2.2), while *t*_0_ was derived from the empirical formula [23]:

log_10_(−*t*_0_) = −0.3922 − 0.2752log_10_
*L*_inf_ − 1.038log_10_*K* (3)

The growth performance index (*ϕ*′) was derived using the specified equation [24]:

*ϕ*′ = log_10_*K* + 2log_10_*L*_inf_ (4)

Fish population dynamics are predominantly influenced by mortality rates, comprising total (*Z*), natural (*M*), and fishing (*F*) components, which satisfy the relationship *Z* = *F* + *M*. The total mortality coefficient (*Z*) was determined through length-converted catch curve analysis with pooled length–frequency data [25] following the equation:

ln(*N_i_*/Δ*t_i_*) = *c* + *dt_i_*′ (5)

where *N_i_* represents the fish count within a specific length class; Δ*t_i_* denotes the duration required for fish growth from the minimum to maximum length in class *i*; *t_i_*′ indicates the age associated with length class *i*; *c* and *d* correspond to the linear equation’s intercept and slope parameters; and *Z* is equal to −*d*.

Natural mortality (*M*) was calculated through Pauly’s empirical formula [25]:

ln*M* = −0.0152 − 0.279ln*L*_inf_ + 0.654ln*K* + 0.463lnT (6)

where T represents the average water temperature in the fish habitat layer, with survey-recorded values of 23.61, 22.91, 27.47, 25.38, 25.26, and 25.94 °C across six consecutive months. The exploitation ratio (*E*) was derived from the *F*/*Z* relationship.

The length at 50% sexual maturity (*L*_50_) was determined through arcsin-square-root (ASR) transformation. For each 5.0 mm size class, the maturity proportion (*P_i_*) was computed. The *L*_50_ value was derived via logistic regression analysis [26]:

(7) $$\mathrm{ASR}(P_{i})\;=\;\frac{\mathrm{ASR}(G)}{1\;+\;\exp[-\delta (X_{i}\;-\;L_{50})]}\;+\;\varepsilon_{i}$$

where *δ* denotes the instantaneous maturation rate, where higher values indicate accelerated maturation; *G* represents the maximum achievable proportion, as all specimens ultimately mature upon attaining critical length; and *ε_i_* constitutes the error term. Nonlinear regression analysis in OriginPro 2021 software generated estimates for *L*_50_, *δ*, and their 95% confidence intervals.

### 2.3. LBB Modeling

This study employed the Length-Based Bayesian Biomass (LBB) model [27] to assess bigeye grunt resource status using exclusively length–frequency data, a method particularly suitable for indeterminately growing fish attaining maximum size at terminal age [28], enabling the estimation of asymptotic length (*L*_inf_), length at first capture (*L*_c_), mortality–growth ratios (*M*/*K* and *F*/*K*), and biomass depletion (*B*/*B*_0_).

The LBB model incorporated the growth dynamics described by the Von Bertalanffy equation (Equation (1)), recommending prioritized use of empirically derived *L*_inf_ values to minimize model uncertainty when such reliable data were available [29].

Following the derivation of *L*_inf_, *L*_c_, *M*/*K*, and *F*/*K* via the LBB model, Equation (8) was applied to compute the maximum unexploited cohort biomass length (*L*_opt_) [30]:

(8) $$L_{\mathrm{opt}}\;={\;L}_{\inf\;}(\frac{3}{3\;+\;M/K})$$

The optimal first-capture length (*L*_c-opt_) was derived using Equation (9) [31]:

(9) $$L_{c\text{-}\mathrm{opt}}\;=\;\frac{L_{\inf}(2\;+\;3F/M)}{(1\;+\;F/M)(3\;+\;M/K)}$$

The yield per recruit (*Y*′/*R*) was determined through Equation (10) [32]:

(10) $$Y^{\prime }/R\;=\;\frac{F/M}{1\;+\;F/M}{(1\;-\;L_{c}/L_{\inf})}^{M/K}(1\;-\;\frac{3(1\;-\;L_{c}/L_{\inf})}{1\;+\;(\frac{1}{M/K\;+\;F/K})}\;+\;\frac{3{(1\;-\;L_{c}/L_{\inf})}^{2}}{\;1\;+\;(\frac{2}{M/K\;+\;F/K})}\;+\;\frac{{(1\;-\;L_{c}/L_{\inf})}^{3}}{1\;+\;(\frac{3}{M/K\;+\;F/K})})$$

The unfished population’s relative biomass (*B*_0_/*R*) was derived through Equation (11) [32]:

(11) $${{B_{0}}^{\prime }/R=\;(1\;-\;L_{c}/L_{\inf})}^{M/K}(1\;-\;\frac{3(1\;-\;L_{c}/L_{\inf})}{1\;+\;(\frac{1}{M/K})}\;+\;\frac{3{(1\;-\;L_{c}/L_{\inf})}^{2}}{1\;+\;(\frac{2}{M/K})}\;+\;\frac{{(1\;-\;L_{c}/L_{\inf})}^{3}}{1\;+\;(\frac{3}{M/K})})$$

where *B*_0_ denotes unfished biomass. And the *B*/*B*_0_ ratio was computed via Equation (12) [32]:

(12) $$B/B_{0}\;=\;\frac{{\mathrm{CPUE}}^{\prime }/R}{{B_{0}}^{\prime }/R}$$

Under the assumption of *F* = *M* and *L*_c_ = *L*_c-opt_, the maximum sustainable yield (MSY) biomass ratio (*B*_msy_/*B*_0_) was determined, with the current-to-MSY biomass ratio (*B*/*B*_msy_) computed via Equation (13) [33]:

(13) $$B/B_{\mathrm{msy}}\;=\;\frac{B/B_{0}}{B_{\mathrm{msy}}/B_{0}}$$

The prior values and LBB parameters for the six years are shown in Table 2.

### 2.4. Generalized Additive Model (GAM)

The GAM can effectively simulate the nonlinear effects of environmental variables on resource abundance. This study employs the GAM to establish the nonlinear response relationships between resource abundance of bigeye grunt in Sierra Leone’s coastal waters and spatial variables as well as environmental variables, thereby analyzing the nonlinear influences of spatial and environmental factors on CPUE. Spatial variables include longitude and latitude, while environmental variables consist of sea surface temperature (SST), deep-sea temperature (DPT), depth, and sea surface salinity (SSS). The GAM formulation used in this study is given below:

log(abundance + 1) ~ *s*(SST) + *s*(DPT) + *s*(longitude) + *s*(latitude) + *s*(depth) + *s*(SSS) + *ε* (14)

where abundance is the response variable resource abundance; *s*(SST) is the environmental explanatory variable sea surface temperature; *s*(DPT) is the environmental explanatory variable deep-sea temperature; *s*(longitude) is the spatial explanatory variable longitude; *s*(latitude) is the spatial explanatory variable latitude; *s*(depth) is the environmental explanatory variable depth; *s*(SSS) is the environmental explanatory variable sea surface salinity; and ε is the random error term.

Collinearity among explanatory variables was assessed using variance inflation factors (VIFs), with detailed results presented in Table 3. All predictors exhibited VIF values below 5, meeting conventional regression diagnostics standards [34,35]. This threshold selection, validated by ecological modeling research [36], effectively balances Type I error control and model simplicity.

Predictor selection was conducted through stepwise regression, employing dual optimization criteria: the Akaike Information Criterion (AIC) [37] and deviance explained [38]. Model adequacy was confirmed by comparative metrics, where superior fits corresponded to reduced AIC scores and elevated explained variance [35,39].

## 3. Results

### 3.1. Spatiotemporal Distribution

**Figure 2:**
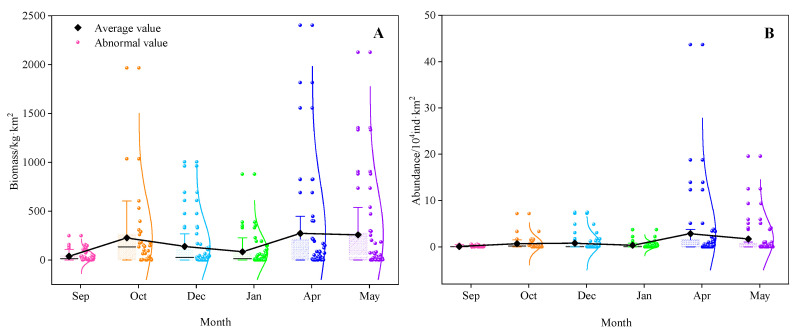
The distribution of biomass (**A**) and abundance (**B**) of bigeye grunt in seasonal bottom trawl surveys.

In these six months, the probability of occurrence of bigeye grunt ranged from 68.29% to 90.32%, and the average occurrence frequency was 82.08% (Table 4). The biomass and abundance of bigeye grunt varied greatly at different stations, and the average value of biomass and abundance varied between 38.18 and 272.39 kg/km^2^ and 1133.24 and 28,597.5 ind/km^2^, respectively, with higher values in April and May and lower values in September and January (Figure 2). The catch of bigeye grunt in the coastal waters of Sierra Leone accounted for 3.33–11.6% by weight and 4.39–34.7% by number.

**Figure 3:**
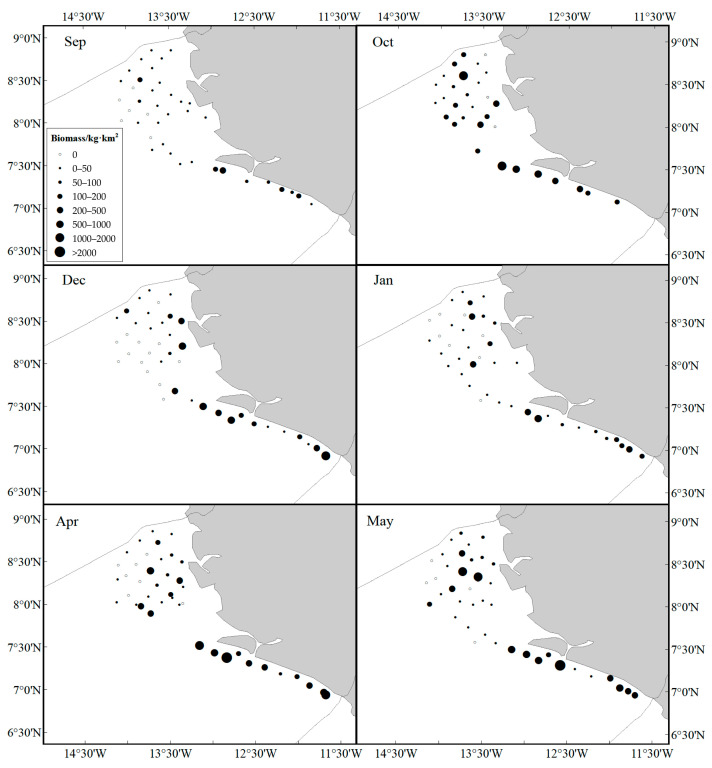
The biomass of bigeye grunt in seasonal bottom trawl surveys.

**Figure 4:**
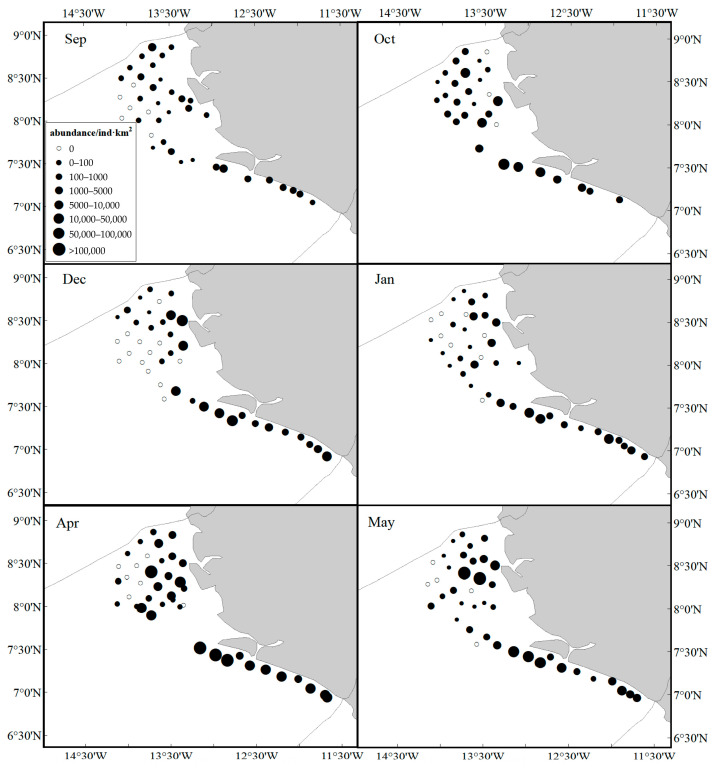
The abundance of bigeye grunt in seasonal bottom trawl surveys.

In September, bigeye grunt was distributed along the continental shelf in the southwest, with a much higher resource biomass south of 7°30′ (Figure 3 and Figure 4). In October, they began to migrate toward the northeast, and the range of large biomass areas began to expand. In December, they were concentrated between 7° N and 7°30′ N, with a smaller distribution area. Just as in December, the higher biomass of bigeye grunt was between 7° N and 7°30′ N in January. The biomass distribution pattern of bigeye grunt was nearly similar in April and May, with a higher biomass than other months and mainly distributed in the southwest.

### 3.2. Variations in Population Parameters

The basic biological characteristics of bigeye grunt, including sex ratio, length range, and mean length, all have showed obvious seasonal characteristics (Table 1). The sex ratio (female to male) of bigeye grunt varied between 0.81 and 1.06. The mean standard length varied from 96.46 mm to 113.03 mm.

Length–weight relationships for bigeye grunt from the coastal waters of Sierra Leone were estimated as follows:

Sep: *W* = 3.72 × 10^−5^*L*^2.94^ (*R*^2^ = 0.9618, n = 851, *p* < 0.001);
Oct: *W* = 3.46 × 10^−5^*L*^2.98^ (*R*^2^ = 0.9735, n = 1062, *p* < 0.001);
Dec: *W* = 6.05 × 10^−5^*L*^2.84^ (*R*^2^ = 0.9836, n = 1001, *p* < 0.001);
Jan: *W* = 2.67 × 10^−5^*L*^3.00^ (*R*^2^ = 0.9802, n = 1236, *p* < 0.001);
Apr: *W* = 1.07 × 10^−5^*L*^3.19^ (*R*^2^ = 0.9866, n = 1231, *p* < 0.001);
May: *W* = 2.45 × 10^−5^*L*^3.02^ (*R*^2^ = 0.9910, n = 1261, *p* < 0.001).

von Bertalanffy growth functions calculated by ELEFAN I are as follows:

Sep: *L_t_* = 172 × (1 − e^−0.34 × (*t* + 0.56)^);
Oct: *L_t_* = 174.8 × (1 − e^−0.29 × (*t* + 0.66)^);
Dec: *L_t_* = 165.5 × (1 − e^−0.40 × (*t* + 0.48)^);
Jan: *L_t_* = 166.2 × (1 − e^−0.34 × (*t* + 0.57)^);
Apr: *L_t_* = 165.1 × (1 − e^−0.38 × (*t* + 0.51)^);
May: *L_t_* = 173.5 × (1 − e^−0.35 × (*t* + 0.54)^).

For length–weight relationships over these six months, the range of *a* was from 1.07 × 10^−5^ (April) to 6.05 × 10^−5^ (December), and *b* was from 2.84 (December) to 3.19 (April) (Table 5). The asymptotic standard length *L*_inf_ varied between 165 mm and 174.8 mm. The *K* values varied slightly, ranging from 0.29 to 0.40, whereas no substantial difference occurred in the *ϕ*′ values (1.91 to 2.03) over time. The *Z* values changed with the season, ranging from 0.57 (October) to 1.57 (September). The values of *M* and *F* varied between 0.44 and 0.60 and 0.13 and 1.07, respectively. The *E* values ranged from 0.22 to 0.49, all of which were below 0.5, suggesting that fishing pressure was unlikely to be the primary driver of the decline in the bigeye grunt population (Table 5).

**Figure 5:**
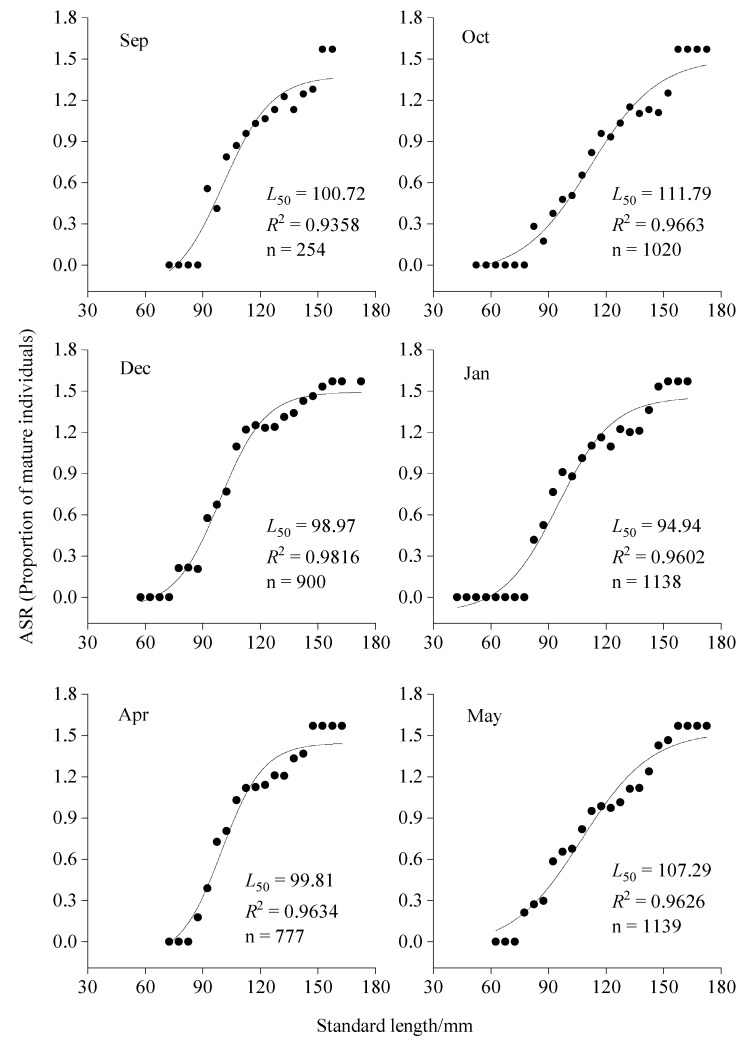
Standard length at the first maturity of bigeye grunt.

Based on an ASR logistic curve fitted by nonlinear regression (Figure 5), the *L*_50_ values of bigeye grunt were obtained for these six months: September, 100.72 mm (95% CI 97.47–103.97 mm); October, 111.79 mm (95% CI 108.65–114.93 mm); December, 98.97 mm (95% CI 97.46–100.48 mm); January, 94.94 mm (95% CI 92.25–97.64 mm); April, 99.81 mm (95% CI 94.82–104.80 mm); May, 107.29 mm (95% CI 103.81–110.77 mm).

### 3.3. Exploitation Status of Bigeye Grunt

**Figure 6:**
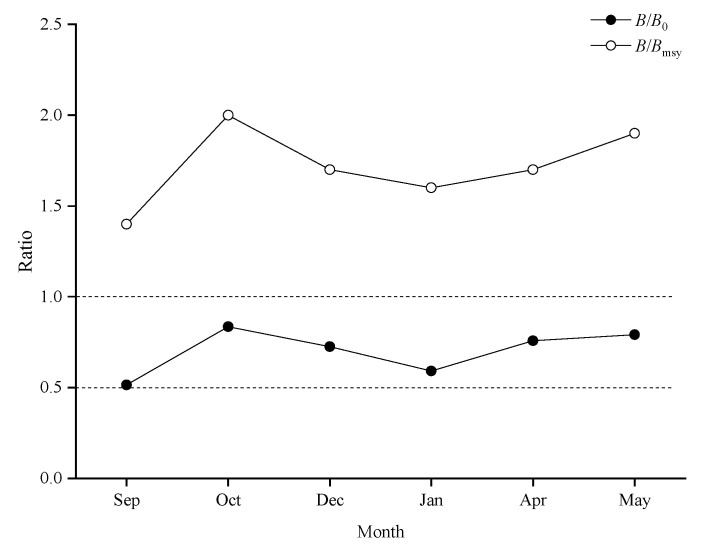
The exploitation status of bigeye grunt.

**Figure 7:**
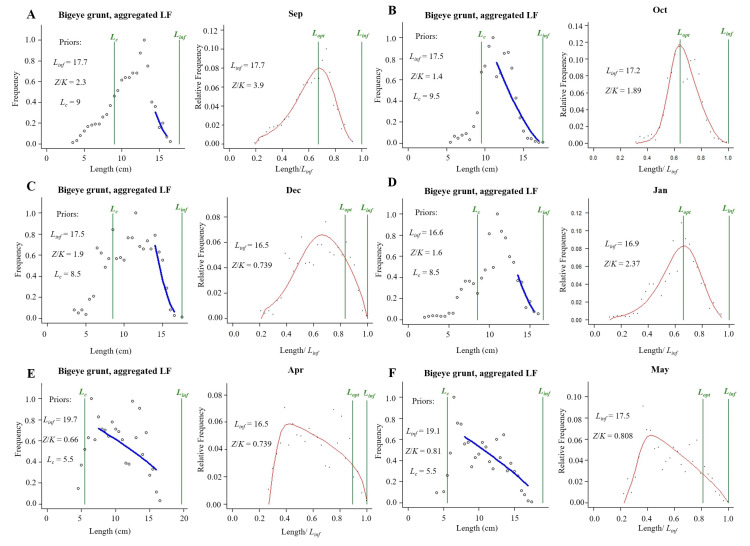
Assessment results of the LBB method for bigeye grunt. Note: (**A**–**F**) represent September, October, December, January, April, and May, respectively. In each subgraph, the upper left panel shows the accumulated length–frequency data used to estimate priors *L*_c_, *L*_inf_, and *Z*/*K*. The right panels show the length–frequency data.

The asymptotic fork length of bigeye grunt varied from 16.5 cm to 17.7 cm (Table 6, Figure 6). No overfishing occurred in the population of this species during the period 2019–2021. The parameters *F*/*M* were all less than 1.0, indicating that the fishing mortality was less than the natural mortality. The parameters *B*/*B*_0_ were greater than 0.5, and *B*/*B*_msy_ were greater than 1, indicating that the bigeye grunt resource is at a high level (Figure 7, Table 6).

### 3.4. GAM Analysis

#### 3.4.1. GAM Test

**Figure 8:**
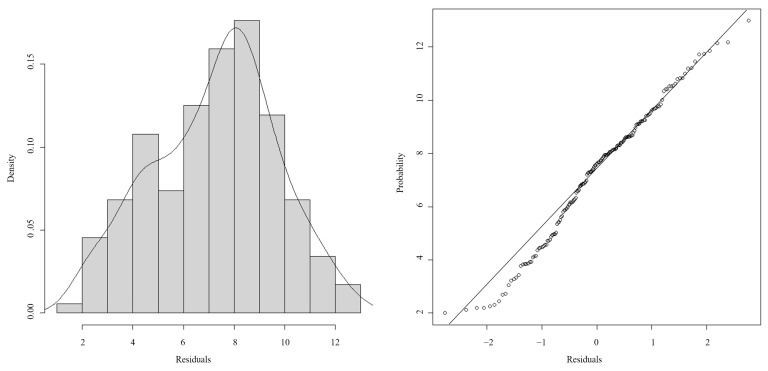
The diagnostic analysis of residuals.

The finalized GAM configuration (Table 7) achieved competitive performance metrics, including an AIC of 730.8089, 53% deviance accounted for, and *R*^2^ = 0.452. Diagnostic evaluation of residuals demonstrated the absence of systematic bias and heteroscedastic variance, concurrent with stabilized effective degree-of-freedom (edf) progression and monotonic AIC reduction, indicative of optimal regularization parameterization (Figure 8). This iterative optimization process ensured structural fidelity to the data distribution without overparameterization. Computational execution was performed via the mgcv package in R 4.4.3.

Table 8 displays the significance levels of predictors in the optimized GAM. F-test outcomes indicated statistically meaningful relationships (*p* < 0.05) between all explanatory variables and the resource abundance.

#### 3.4.2. Distribution of Abundance Under Different Factors

**Figure 9:**
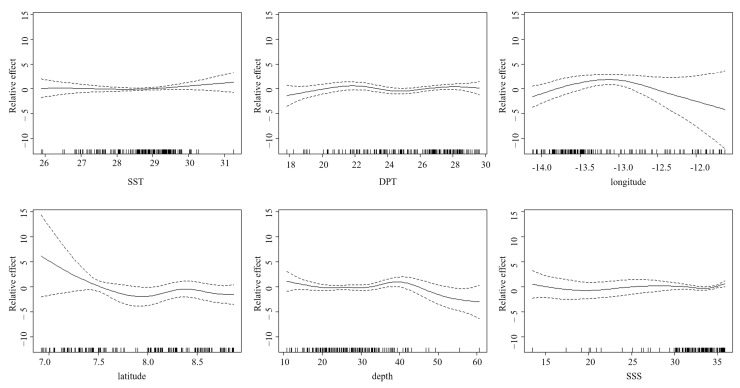
The impact of each explanatory variable on the abundance of bigeye grunt.

Among the explanatory variables, latitude exhibited the highest level of influence, accounting for 27.1% of the deviance explained, while depth showed the lowest impact, explaining merely 3.8% of the deviance (Table 4). Figure 9 demonstrates that all variables exhibit nonlinear effects on the resource abundance of bigeye grunt. The impact of SST on abundance showed a trend of being stable first and then changing. When the SST was between 26 and 29 °C, the curve was close to the 0 value, indicating that this temperature range has little impact on the resource abundance offset of the bigeye grunt; when it was higher than 29 °C, the impact on resource abundance began to differentiate. As the DPT increased, the abundance was generally relatively stable. There was a relatively obvious upward trend when the DPT was between 18 and 22 °C, and thereafter, the impact of the DPT was no longer significant. The abundance showed a trend of first increasing and then decreasing with the increase in longitude, reaching the maximum value at 13.1° W. With the increase in latitude, the abundance showed a decreasing trend, indicating that the resource abundance of the bigeye grunt is greater in the southern coastal waters of Sierra Leone than in the northern coastal waters. When the depth was between 10 and 33 m, the resource abundance shows a relatively stable trend, and then it gradually increased, reaching the maximum value at 40 m. When the depth exceeded 40 m, the resource abundance decreased rapidly. With the change in SSS, the change in abundance was relatively gentle overall, but when the salinity was greater than 33, the abundance had a significant increasing trend.

## 4. Discussion

The bigeye grunt is among the most abundant species along the coast of Sierra Leone [40]. Despite its ubiquitous occurrence in Sierra Leonean littoral zones, this species exhibits significant spatial and seasonal variations in population biomass and abundance (Figure 2 and Figure 3). These results may be influenced by multiple habitat factors, such as water temperature, salinity, food availability, and reproductive cycles, which collectively govern the spawning, growth, and migratory behaviors of bigeye grunt. GAM analysis shows that the resource abundance has certain nonlinear relationships with SST, DPT, longitude, latitude, depth, and SSS, among which latitude has the greatest impact (Figure 9). This result confirms the importance of environmental factors and geographical location factors in the habitat of bigeye grunt. Sierra Leone experiences a distinct tropical monsoon climate characterized by pronounced wet and dry seasons annually, with minimal temperature variation but significant salinity fluctuations [11]. Research indicates that salinity may not only influence bigeye grunt abundance but also serve as a key regulator of its growth, development, and reproduction [8]. Comparatively, abundance exhibited more pronounced seasonal amplitude than biomass. Our results demonstrate statistically significant variations in the biomass and abundance of bigeye grunt. Comparative analysis revealed that the latter’s mean values exhibited substantially greater fluctuations than the former. It is worth noting that although the peaks of both indicators occur in April, the abundance in April is significantly higher than that in all other months, while the biomass in April shows little difference from that in May (Figure 4). This divergence between biomass and abundance patterns is inextricably linked to their reproductive biology. We postulate that substantial April and May spawning events in bigeye grunt drive pronounced abundance surges, while limited biomass increases stem from the low biomass of recruiting cohorts. Therefore, we consider April to be the peak breeding period of bigeye grunt, with breeding activities starting to decline in May, and its breeding period should mainly occur during the transition from the dry season to the rainy season. Zhao et al. [9] similarly concluded that April and May likely represent the spawning period for bigeye grunt, a reproductive pattern consistent with most dominant coastal species in Sierra Leone, where peak spawning occurs during the dry season or its transition to the wet season [41,42]. Although May marks the start of the rainy season in Sierra Leone, rainfall remains scarce, solar radiation is relatively high, and the marine environmental characteristics are still similar to those of the dry season. This phenomenon is known as the “lag effect,” whose duration often depends on the seasonal cycles in the climate [40]. Therefore, the duration of this “lag effect” is likely to have a significant impact on the reproduction of bigeye grunt. In addition, spatial variations in fishing pressure may additionally shape distribution patterns. Although this study does not directly assess spatial heterogeneity in fishing intensity, historical data suggest that anthropogenic disturbances near traditional fishing grounds may indirectly influence population aggregation dynamics [43,44,45,46].

Analysis of bigeye grunt growth parameters across monthly intervals revealed consistently low exploitation rates (*E*-values < 0.5) (Table 7), indicating no current overfishing pressure on this species in the coastal waters of Sierra Leone. These conclusions are corroborated by LBB method assessment results, demonstrating that fishing mortality remains below natural mortality, while biomass persists at sustainable levels. Collectively, this evidence supports the existence of sustainable exploitation potential for bigeye grunt populations in the coastal waters of Sierra Leone. Feng et al. [40] demonstrated that bigeye grunt is the species contributing the most to the abundance of coastal fishery resources in Sierra Leone, with the highest Index of Relative Importance (IRI). This finding further confirms that bigeye grunt has a high resource abundance in the coastal waters of Sierra Leone. Notably, although CECAF has repeatedly indicated overexploitation of this species throughout most of its distribution range in the Eastern Central Atlantic [1], this study provides the first localized dataset from Sierra Leone waters, revealing marked inter-regional variations in stock status. This outcome not only challenges CECAF’s prior assessment conclusions regarding the species but also emphasizes the imperative of incorporating regional variability into fishery stock assessments. Indeed, research on resource assessments of bigeye grunt in West African coastal waters has been severely lacking over the past decade, with the limited existing evaluations conducted over ten years ago [6,47,48]. This research gap thereby underscores the crucial significance and necessity of the present study.

This study pioneers the application of the LBB method to stock assessment of bigeye grunt, with the results cross-validated against conventional growth parameters. The demonstrated scientific merit of this approach lies in its capacity to serve as an innovative solution for data-limited marine fishery assessments [28]. Having gained widespread adoption, the LBB model has proven robust in practice by not only eliminating traditional methods’ dependence on large datasets but also accurately characterizing fish growth and mortality patterns [49]. These capabilities provide substantial support for developing science-based management strategies. However, persistent challenges including model parameter sensitivity and data quality uncertainties require further investigation and refinement in future research [50].

This study addresses critical knowledge gaps regarding bigeye grunt stock assessments in Sierra Leonean waters, significantly enhancing our understanding of the species’ status throughout the Eastern Central Atlantic. We not only visualized the distribution of bigeye grunt in the coastal waters of Sierra Leone but also conducted a resource assessment and explored the relationship between its resource abundance and key environmental factors as well as geographical locations. These findings provide crucial scientific foundations for regional fisheries management, prompting the following recommendations: (1) implement a dynamic stock monitoring system to track population dynamics of key fishery resources like bigeye grunt in real time, enabling timely management adjustments; (2) enhance fisheries research to improve assessment accuracy and reliability; and (3) foster collaborative frameworks between fisheries management authorities and research institutions to develop science-based policies ensuring responsible resource utilization.

## 5. Conclusions

This study is the first to fully assess bigeye grunt populations in the coastal waters of Sierra Leone, improving regional resource knowledge and supporting science-based management in the Eastern Central Atlantic. The results show that bigeye grunt is widely distributed but with varying biomass and abundance over space and time, linked to environmental factors, reproduction, and human activities. Regarding growth parameters and exploitation status, bigeye grunt exhibited statistically significant monthly variations in growth characteristics. However, all calculated exploitation rates (*E*) remained consistently below the 0.5 threshold. Combined with LBB estimation analyses, these results collectively demonstrate the absence of overfishing for bigeye grunt populations in the coastal waters of Sierra Leone, indicating sustainable exploitation potential. This finding presents a stark contrast to CECAF’s prior assessment of widespread overexploitation across most Eastern Central Atlantic distribution ranges, highlighting the distinct stock status in Sierra Leonean waters. In addition, there is a certain nonlinear relationship between the resource abundance and environmental factors as well as geographical locations, among which latitude has the greatest impact, while depth has the smallest. The analysis suggests that the peak breeding period of bigeye grunt occurs in April, specifically during the transition from the dry season to the rainy season.

Our findings show that bigeye grunt stocks in Sierra Leone’s coastal waters can be sustainably managed. This study supports science-based fishing strategies, suggesting maintaining current catch levels while improving population monitoring. Applying ecosystem-based fisheries principles would help develop conservation measures for long-term resource sustainability.

## Figures and Tables

**Table 1:** The sample of bigeye grunt.

Month	Total Individuals	Sex Ratio (♀/♂)	Standard Length Range (mm)	Mean Standard Length ± SD (mm)
Sep	851	0.89	30~162	107.95 ± 26.67
Oct	1062	0.81	54~172	113.03 ± 18.55
Dec	1001	0.99	32~164	105.23 ± 28.70
Jan	1236	0.87	20~164	104.1 ± 28.41
Apr	1231	0.82	40~163	98.19 ± 30.47
May	1261	1.06	38~173	96.46 ± 31.44

**Table 2:** Scenarios and priors of bigeye grunt.

Month	Class Bin (mm)	*L*_inf_ Prior	*Z*/*K* Prior	*M*/*K* Prior	*F*/*K* Prior	*L*_c_ Prior	Alpha Prior
Sep	5	16.6	1.74	1.5	0.235	8.67	8.69
Oct	5	17.5	1.42	1.5	0.3	9.69	32.5
Dec	5	17.5	1.93	1.5	0.428	8.67	9.86
Jan	5	16.6	1.64	1.5	0.138	8.67	10.4
Apr	5	19.7	0.663	1.5	0.3	5.61	37.5
May	5	19.1	0.808	1.5	0.3	5.61	26.9

Note: *L*_inf_, asymptotic length; *K*, growth rate; *Z*, total mortality; *M*, natural mortality; *F*, fishing mortality; *L*_c_, length at first capture.

**Table 3:** Variable VIF testing results.

Variable	SST	DPT	Longitude	Latitude	Depth	SSS
VIF value	1.860	2.083	4.138	4.074	1.657	1.421

Note: SST, sea surface temperature; DPT, deep-sea temperature; SSS, sea surface salinity.

**Table 4:** Probability of occurrence, contributions, average biomass and abundance of bigeye grunt in bottom trawl surveys.

Month	Probability of Occurrence (%)	Average Biomass ± SE (kg/km^2^)	Average Abundance ± SE (ind/km^2^)	Contribution to the Total Weight (%)	Contribution to the Total Number (%)
Sep	84.62	38.18 ± 8.74	1133.24 ± 241.77	3.33	4.39
Oct	90.32	228.18 ± 70.38	6998.36 ± 2520.58	11.6	20.29
Dec	68.29	139.73 ± 39.92	7819.81 ± 2779.11	7.93	21.8
Jan	80.95	84.32 ± 24.85	3434.13 ± 1070.49	4.87	11.65
Apr	82.93	272.39 ± 82.10	28,597.5 ± 11,969.11	10.17	34.7
May	85.37	258.6 ± 76.67	17,057.22 ± 6241.67	7.4	24.11

**Table 5:** Variations in growth and mortality parameters of bigeye grunt.

Month	Sep	Oct	Dec	Jan	Apr	May
*a*	3.72 × 10^−5^	3.46 × 10^−5^	6.05 × 10^−5^	2.67 × 10^−5^	1.07 × 10^−5^	2.45 × 10^−5^
*b*	2.94	2.98	2.84	3.00	3.19	3.03
*L*_inf_	165	174.8	165.5	166.2	165.1	173.5
*K*	0.30	0.29	0.40	0.34	0.38	0.35
*Z*	0.91	0.57	1.11	0.68	1.09	0.98
*M*	0.47	0.44	0.60	0.52	0.56	0.53
*F*	0.44	0.13	0.51	0.16	0.53	0.45
*E*	0.49	0.22	0.46	0.23	0.48	0.46
*t*_0_	−0.65	−0.66	−0.48	−0.57	−0.51	−0.54
*ϕ*′	1.91	1.95	2.03	1.97	2.01	2.02
*L*_50_	100.72	111.79	98.97	94.94	99.81	107.29

**Table 6:** Summary of the LBB model results.

Month	Sep	Oct	Dec	Jan	Apr	May
*L*_inf_	16.7	17.2	16.5	16.9	16.5	17.5
*L*_opt_	11	11	14	11	15	14
*L*_c-opt_	9.3	7.8	10	8.7	11	10
*M*/*K*	1.44	1.66	0.585	1.54	0.352	0.699
*F*/*K*	1.3	0.226	0.154	0.831	0.0766	0.109
*Z*/*K*	2.7	1.9	0.747	2.36	0.431	0.809
*F*/*M*	0.908	0.136	0.263	0.532	0.22	0.158
*B*/*B*_0_	0.514	0.835	0.725	0.591	0.758	0.791
*B*/*B*_msy_	1.4	2.0	1.7	1.6	1.7	1.9
*L*_c_	12.9	10.1	8.84	6.61	5.23	5.7
*L*_95_th/*L*_inf_	0.98	0.99	1	1	1	1
Status	healthy	healthy	healthy	healthy	healthy	healthy

**Table 7:** GAM selection based on AIC.

GAM	*R* ^2^	AIC	Explanation Rate (%)
log(abundance) ~ *s*(SST)	0.0357	809.4270	4.71
log(abundance) ~ *s*(SST) + *s*(DPT)	0.0861	803.7746	11.8
log(abundance) ~ *s*(SST) + *s*(DPT) + *s*(longitude)	0.357	744.7764	38.9
log(abundance) ~ *s*(SST) + *s*(DPT) + *s*(longitude) + *s*(latitude)	0.402	736.9369	45.1
log(abundance) ~ *s*(SST) + *s*(DPT) + *s*(longitude) + *s*(latitude) + *s*(depth)	0.429	732.3952	48.9
log(abundance) ~ *s*(SST) + *s*(DPT) + *s*(longitude) + *s*(latitude) + *s*(depth) + *s*(SSS)	0.452	730.8089	53

**Table 8:** ANOVA of the optimal GAM.

Parameter	df	F	*p* Value
SST	2.085	2.585	0.0392
DPT	4.004	2.402	0.0413
longitude	4.187	16.3	2 × 10^−16^
latitude	6.167	7.691	2 × 10^−16^
depth	6.857	3.029	0.00304
SSS	1	0.663	2 × 10^−16^

## Data Availability

The original contributions presented in the study are included in the article, and further inquiries can be directed to the corresponding author.
